# Social isolation, mental health, and use of digital interventions in youth during the COVID-19 pandemic: A nationally representative survey

**DOI:** 10.1192/j.eurpsy.2021.17

**Published:** 2021-03-09

**Authors:** Christian Rauschenberg, Anita Schick, Christian Goetzl, Susanne Roehr, Steffi G. Riedel-Heller, Georgia Koppe, Daniel Durstewitz, Silvia Krumm, Ulrich Reininghaus

**Affiliations:** 1 Department of Public Mental Health, Central Institute of Mental Health, Medical Faculty Mannheim, Heidelberg University, Mannheim, Germany; 2 Department of Psychiatry and Neuropsychology, School for Mental Health and Neuroscience, Maastricht University, Maastricht, The Netherlands; 3 Department of Psychiatry II, University of Ulm and BKH Guenzburg, Ulm, Germany; 4 Institute of Social Medicine, Occupational Health and Public Health (ISAP), Medical Faculty, University of Leipzig, Leipzig, Germany; 5 Department of Theoretical Neuroscience, Central Institute of Mental Health, Medical Faculty Mannheim, Heidelberg University, Mannheim, Germany; 6 Department of Psychiatry and Psychotherapy, Central Institute of Mental Health, Medical Faculty Mannheim, Heidelberg University, Mannheim, Germany; 7 Centre for Epidemiology and Public Health, Health Service and Population Research Department, Institute of Psychiatry, Psychology & Neuroscience, King’s College London, London, United Kingdom; 8 ESRC Centre for Society and Mental Health, King’s College London, London, United Kingdom

**Keywords:** COVID-19, mHealth, Social isolation, Social risk, Youth mental health

## Abstract

**Background:**

Public health measures to curb SARS-CoV-2 transmission rates may have negative psychosocial consequences in youth. Digital interventions may help to mitigate these effects. We investigated the associations between social isolation, COVID-19-related cognitive preoccupation, worries, and anxiety, objective social risk indicators, and psychological distress, as well as use of, and attitude toward, mobile health (mHealth) interventions in youth.

**Methods:**

Data were collected as part of the “Mental Health And Innovation During COVID-19 Survey”—a cross-sectional panel study including a representative sample of individuals aged 16–25 years (*N* = 666; M_age_ = 21.3; assessment period: May 5, 2020 to May 16, 2020).

**Results:**

Overall, 38% of youth met criteria for moderate or severe psychological distress. Social isolation worries and anxiety, and objective risk indicators were associated with psychological distress, with evidence of dose–response relationships for some of these associations. For instance, psychological distress was progressively more likely to occur as levels of social isolation increased (reporting “never” as reference group: “occasionally”: adjusted odds ratio [aOR] 9.1, 95% confidence interval [CI] 4.3–19.1, *p* < 0.001; “often”: aOR 22.2, CI 9.8–50.2, *p* < 0.001; “very often”: aOR 42.3, CI 14.1–126.8, *p* < 0.001). There was evidence that psychological distress, worries, and anxiety were associated with a positive attitude toward using mHealth interventions, whereas psychological distress, worries, and anxiety were associated with actual use.

**Conclusions:**

Public health measures during pandemics may be associated with poor mental health outcomes in youth. Evidence-based digital interventions may help mitigate the negative psychosocial impact without risk of viral infection given there is an objective need and subjective demand.

## Introduction

As of March 2020, most European countries have adopted a range of public health measures to lower the transmission of SARS-CoV-2 coronavirus. Physical distancing and quarantine have been among the most important non-pharmacological measures to reduce infection rates of coronavirus disease 2019 (COVID-19). These preventive measures, however, may have a profound impact on public mental health. Studies investigating the psychosocial impact of earlier pandemics (e.g., SARS and MERS) have shown that physical distancing and quarantine have immediate as well as prolonged effects on individuals’ mental health, including depression, anxiety, psychosis, and perceived stress [1–5]. Furthermore, it has been found that these safety measures are associated with an increase of more distal risk factors for poor mental health such as social isolation, risk behaviors (e.g., cannabis and alcohol misuse), and lowered physical activity [1]. In line with findings on earlier outbreaks, accumulating evidence suggests negative psychosocial consequences of the current COVID-19 pandemic on public mental health, including increased levels of depression, anxiety, self-harm, and loneliness [6–27]. Although the increasing number of approved vaccines and potential breakthroughs in the pharmacological treatment of COVID-19 are reasons for optimism, health-related outcomes may worsen at any time due to new virus variants (e.g., lineage B.1.1.7 or B.1.351) as well as economic uncertainties and recession, which may occur secondary to the pandemic.

There is also evidence that the detrimental effects of pandemics are disproportionally distributed across communities: societal inequalities have been found to increase the risk of COVID-19 on various health domains. Those with inferior social position, for instance, have been found to have increased disease fatality and hospital admission rates as well as to experience more severe psychosocial and economic consequences [28], and initial findings from the United Kingdom suggest inequalities in adverse experiences during the early weeks of the lockdown [29]. Other studies have found that individuals with histories of migration and unemployment experience more severe depressive and anxiety symptoms, especially in youth [30].

Information and communication technologies may be particularly important in alleviating COVID-19-related psychosocial consequences [31]. For instance, smartphone applications (apps) help individuals to remotely interact with others (e.g., by using videoconferencing software) and digital interventions, which do not require face-to-face contact (e.g., Internet-based interventions [eHealth] and mobile health applications [mHealth apps]), may help to increase public mental health during health crises [31]. Previous studies have shown that digital tools available in major app stores, especially mHealth apps, are already frequently being used, although most developers do not provide information on their evidence base, safety, and effectiveness [32–34]. While, in contrast, eHealth and mHealth interventions that have been developed and evaluated by research groups signal great promise on their safety, acceptability, and effectiveness across the whole spectrum of public mental health provision (i.e., mental health promotion, prevention, and treatment of mental disorders), especially if embedded in social and therapeutic contexts (e.g., peer-support and blended care) [31,35]. Thus, although mHealth apps available in app stores should be used with caution, digital interventions may be used to mitigate the negative impact of the COVID-19 pandemic [31]. If used purposefully, these tools may help to provide low-threshold, timely, and personalized public mental health care and can be tailored to the individual needs—even under the restrictive conditions of the COVID-19 pandemic and without the risk of viral infection [31,36–38]. However, to the best of our knowledge, there has been no study to date which has specifically investigated the role of publicly available mHealth apps during public health crises, including the current COVID-19 pandemic, and the available evidence on the occurrence of psychological distress in young individuals and important correlates remain very limited.

In the current study, we aimed to investigate the associations between social isolation, COVID-19-related cognitive preoccupation, worries, and anxiety, objective social risk indicators, and psychological distress, as well as use of, and attitude toward, digital mHealth apps in a representative sample of youth aged 16–25 from the general population. Data collection took place in May 2020 and during active lockdown in Germany. Specifically, we sought to test the following hypotheses: First, (a) social isolation and lack of company, (b) COVID-19-related cognitive preoccupation, worries, and anxiety, and (c) objective indicators of social risk (e.g., unemployment, migrant or ethnic minority group position) are associated with occurrence of psychological distress. Second, these associations are consistent with a dose–response pattern. Third, current use of, and positive attitudes toward, mHealth apps are more common in those who experience psychological distress, more frequent social isolation and lack of company, COVID-19-related preoccupation, worries, and anxiety, and who are exposed to more objective indicators of social risk.

## Methods

### Design and participants

Data were drawn from the “Mental Health And Innovation During COVID-19 Survey”—a cross-sectional panel study. This study was conducted as part of a living lab entitled “AI4U—Artificial Intelligence for personalized digital mental health promotion and prevention in youth,” which aims to develop, optimize, evaluate, and implement digital artificial intelligence-based interventions in routine public mental health provision by adopting a transdisciplinary approach involving users from the target population and relevant stakeholders in all stages of the research process. We recruited a representative sample of youth aged 16–25 from the German general population. The study commenced on May 7, 2020 and was completed on May 16, 2020. Thus, data were collected at times of active lockdown measures to lower transmission rates. More specifically, during this time period, region-specific measures were enacted to prevent the spread of SARS-CoV-2, including the closure of schools, kindergartens, playgrounds, zoos, churches, sports clubs, services that require close physical contact (e.g., hairdressers), and nonessential shops. In addition, it was forbidden to leave the house without a good reason, and it was only allowed to have contact with one other person not living in the same household. Furthermore, keeping a physical distance of 1.5 m and wearing face masks in public places as well as in public transportation was obligatory. Also, in order to reduce the effects of these measures on the population and the economy, many companies received state aid to be able to pay for running costs (e.g., personnel costs and rent).

For data collection, we used the Norstatpanel by Norstat Deutschland GmbH [39], which consists of a group of registered internet users who have agreed to take part in surveys and opinion polls and is certified according to ISO 26362 and ISO 9001 standards. To ensure the high quality of the panel, various quality assurance measures have been implemented and are frequently evaluated, such as random selection, representativeness, diversified sources, and active recruitment of panelists, as well as the absence of a public registration page, profile validation, plausibility testing, and cheater detection. The online panel operates in accordance with the applicable data protection laws (i.e., EU General Data Protection Regulation and Federal Data Protection Act). Prior to assessments, informed consent was obtained from participants in this general population sample. Participants were registered members of the Norstatpanel and selected at random. Selected individuals were invited by email to participate in the online survey. To ensure representativeness of the sample, individuals were stratified by gender, education, and population density data published by the Federal Statistical Office of Germany. Participation was incentivized through payments (i.e., around 0.10€ per minute) and other benefits (e.g., discounts). All procedures performed in studies involving human participants were in accordance with the ethical standards of the institutional and/or national research committee and with the 1964 Helsinki Declaration and its latter amendments or comparable ethical standards. The study was approved by the Medical Ethics Review Committee II of Heidelberg University (Medical Faculty Mannheim; Ref. No. 529-20).

### Measures

#### Social isolation/lack of company

Social isolation and lack of company were assessed using two items of the Three-Item Loneliness Scale, which has been developed based on the 20-item Revised UCLA Loneliness Scale [40] and was specifically developed to assess loneliness in large-scale surveys [41]. However, as we were interested in measuring social isolation and lack of company, we excluded one item assessing the feeling of being left out. Subjective experiences of social isolation (“*How often do you feel socially isolated?*”) and lack of company (“*How often do you feel that you lack the company of others?*”) were both rated on a 6-point Likert scale ranging from 1 to 6 (1 = “never,” 3 = “rarely,” 6 = “very often”). A high internal consistency has been demonstrated for different versions of the UCLA Loneliness Scale, including the Three-Item Loneliness Scale [41,42].

#### COVID-19-related cognitive preoccupation, worries, and anxiety

COVID-19-related cognitive preoccupation, worries, and anxiety were assessed using modified items from the COVID-19 Snapshot MOnitoring (COSMO) [30] survey in Germany. First, worries were assessed using 10 items introduced by the following sentence: *“On a scale from 1 (no worries at all) to 7 (a lot of worries), how often did you worry last week that…,”* which was followed by differing types of worries (e.g., about financial difficulties). For current analyses, we computed the overall mean score (Cronbach’s alpha, α = 0.80). We also dichotomized the continuous score using median split of the continuous variable: <50th percentile was coded as 0 and ≥50th percentile was coded as 1). Second, preoccupation and anxiety with the COVID-19 pandemic were assessed using three separate items rated on a 5-point scale (*“The novel coronavirus is something I…*,” with ratings ranging from 1 *“…never think of”* to 5 *“…keep thinking about”*; *“The novel coronavirus is…,”* with ratings ranging from 1 *“…not scary at all”* to 5 *“…scary”*; “*The novel coronavirus is…,”* with ratings ranging from 1 *“…not worrying”* to 5 *“…worrying”*).

#### Objective social risk indicators

Data on objective indicators of individuals’ social circumstances and migrant/ethnic minority group position were assessed using a modified version of the Medical Research Council Sociodemographic Schedule [43]. In total, six domains of social risk were included in the current study: (a) employment, (b) education, (c) relationship status, (d) living arrangements, (e) parental educational level as a proxy for lower socioeconomic status, and (f) migrant or ethnic minority group position. To investigate the impact of social risk, we built on the work by Morgan et al. [44], and created an index by dichotomizing variables from each of the six domains to define the presence or absence of well-established indicators of social risk (i.e., [a] unemployment, unable to work, early retirement = 1, other = 0; [b] lower educational level [i.e., secondary school and no school-leaving qualification] = 1, other = 0; [c] being single = 1, other = 0; [d] living alone or alone with children = 1, other = 0; [e] lower parental educational level as a proxy for lower socioeconomic status [i.e., secondary school and no school-leaving qualification of both parents] = 1, other = 0; [f] foreign born or second generation migrant = 1, other = 0). This generated an index ranging from 0 to 6.

#### Current use of, and attitudes toward, mHealth apps

After providing a definition of mHealth apps, participants were asked whether they are already using mHealth apps by asking the following question *“Do you already use mHealth apps (e.g., to relax or increase physical activity)?”* This item was rated on a 6-point Likert scale and dichotomized (a rating of 1 = “never” was coded as 0, and ratings of 2 = “very rarely,” and 4 = “occasionally,” to 6 = “very often” were coded as 1). This item was followed by an item assessing the positive, negative, or neutral attitude toward the use of mHealth apps to help cope with the COVID-19 pandemic *(“Do you think that an mHealth app could help you deal better with the corona situation?”)*. This item was rated on a 5-point Likert scale and dichotomized (with ratings of 1 = “strongly disagree” to 3 = “neither/nor” were coded as 0, and ratings of 4 = “agree” and 5 = “strongly agree” were coded as 1).

#### Psychological distress

The Kessler-10 (K10) [45], a well-established screening instrument for mental disorder in the general population, was used to assess psychological distress. The questionnaire was modified to assess psychological distress during the COVID-19 pandemic. That is, instead of asking about psychological distress experienced in the past 30 days, psychological distress experienced since the beginning of the pandemic was assessed (“*How often did you feel since the coronavirus outbreak…”* followed, e.g., by *“…tired out for no good reason?”*). The 10 items were rated on a 5-point scale (1 = “none of the time,” 3 = “some of the time,” and 5 = “all of the time”), yielding a minimum score of 10 and a maximum score of 50 (Cronbach’s alpha, α = 0.93). For analyses including psychological distress as dependent variable, we dichotomized continuous distress scores based on an established cutoff score (absence of psychological distress: scores from 10 to 19 were coded as 0; presence of mild, moderate or severe psychological distress: scores from 20 to 50 were coded as 1). Good psychometric properties have been reported for this measure [46]. For analyses in which psychological distress was used as independent variable, we used a categorical variable with four levels: (a) likely to be well (range score: 10–19), (b) mild mental disorder (range score: 20–24), (c) moderate mental disorder (range score: 25–29), and (d) severe mental disorder (range score: 30–50), again, based on established and validated cutoff scores [45,47].

## Analysis

Descriptive statistics were used to report on basic sample characteristics. Logistic regression was used to, first, quantify the association of (a) social isolation/lack of company, (b) COVID-19-related cognitive preoccupation, worrying, and anxiety, and (c) objective indicators of social risk (separate as well as combined in a form of a social risk index) as independent variables with psychological distress as outcome variable (hypothesis a). This approach allows for examining dose–response relationships (hypothesis b). Second, we investigated whether (a) psychological distress, (b) social isolation/lack of company, (c) COVID-19-related preoccupation, worries, and anxiety, and (d) the objective indicators of social risk as well as the social risk index are associated with the current use of, and attitude toward, mHealth apps (hypothesis c). In all analyses, we adjusted for potential confounders (i.e., age, gender, educational level, migrant/ethnic minority group position, and employment status), except in models that included social risk indicators as independent variable. Here, we adjusted for age and gender. We adjusted significance levels for Type-1 error proliferation using family-wise error-corrected *p*-values (*p*FWE) by multiplying the unadjusted *p*-value by the total number of independent variables (*N* = 7 for models with psychological distress as dependent variable; *N* = 8 for models with the use of, or attitude toward, mHealth apps as dependent variable). All analyses were performed using STATA version 15.1.

## Results

### Sample characteristics

In total, 1006 individuals were invited by email to participate. Of these, 685 youths completed the online survey and 19 individuals had to be excluded after completion of quality control checks (e.g., implausible response time and pattern of responses). Thus, 666 individuals were included in current analyses. There were no differences in variables between individuals with and without sufficient data quality (data not shown, available upon request). The sample characteristics are presented in Table 1, including frequencies of all assessed variables.

**Table 1.** Sample characteristics, psychological distress, and risk (*N* = 666).

Age, mean (SD; range)	21.3 (2.6; 16–25)
Gender, *n* (%)	
Female	318 (47.8)
Male	346 (52.0)
Divers	2 (0.2)
Educational level, *n* (%)^a^	
Low	135 (20.3)
Middle	358 (53.7)
High	173 (26.0)
Migrant/ethnic minority group position, *n* (%)	
First generation migrant	53 (7.9)
Second generation migrant	156 (23.4)
Psychological distress,^b^ *n* (%)	
Likely to be well	287 (43.1)
Likely to have a mild disorder	130 (19.5)
Likely to have a moderate disorder	104 (15.6)
Likely to have a severe disorder	145 (21.8)
Psychological distress, mean (SD; range)	22.0 (8.6; 10–50)
Social isolation, *n* (%)	
Never	61 (9.2)
Very rarely	65 (9.8)
Rarely	135 (20.3)
Occasionally	212 (31.8)
Often	136 (20.4)
Very often	57 (8.6)
Lack of company, *n* (%)	
Never	34 (5.1)
Very rarely	43 (6.5)
Rarely	81 (12.2)
Occasionally	245 (36.8)
Often	178 (26.7)
Very often	85 (12.3)
COVID-19-related worries,^c^ *n* (%)	
<50th percentile	350 (52.6)
≥50th percentile	316 (47.4)
Mean (SD; range)	3.6 (1.2; 1–7)
Cognitive preoccupation with COVID-19	
Item: “*The novel coronavirus is something I…,” n* (%)	
…never think of	20 (3.0)
…do not think about very often	115 (17.3)
…think in part	245 (36.8)
…think about a lot	245 (36.8)
…keep thinking about	41 (6.2)
COVID-19-related anxiety	
Item: *“The novel coronavirus is…*,” *n* (%)	
…not scary at all	105 (15.8)
…rather not scary	187 (28.1)
…partly scary	219 (32.9)
…rather scary	133 (20.0)
…scary	22 (3.3)
COVID-19-related worrying	
Item*: “The novel coronavirus is…*,” *n* (%)	
…not worrying	70 (10.5)
…rather not worrying	142 (21.3)
…partly worrying	207 (31.1)
…rather worrying	207 (31.1)
…worrying	40 (6.0)
Social risk index^d^	
0	160 (24.0)
1	270 (40.5)
2	174 (26.1)
3+	62 (9.3)
Current use of mHealth apps^e^	
Yes	473 (71.0)
No	193 (29.0)
Attitude toward the use of mHealth apps during the COVID-19 pandemic^f^	
Positive	170 (25.5)
Neutral/negative	496 (74.5)

Abbreviation: SD, standard deviation.

a Educational levels were defined as follows: “low” (i.e., lower secondary school certificate, secondary school certificate, no school-leaving qualification, or visiting respective school types); “middle” (i.e., high-school diploma, completed vocational training, or visiting respective school type/doing an apprenticeship); “high” (i.e., bachelor’s degree, master’s degree, or currently studying).

b The following K10 cutoffs were used to categorize severity levels of psychological distress: “none” (range score: 10–19); “mild” (range score: 20–24); “moderate” (range score: 25–29); “severe” (range score: 30–50).

c Based on 10 items asking for potential worries related to the COVID-19 pandemic (e.g., health system overloaded, financial difficulties, and completion of education/school) during the last week rated on a 7-point Likert scale (1 = “no worries at all”; 7 = “a lot of worries”). Items were dichotomized (i.e., median split of the continues variable: <50th percentile was coded as 0 and ≥50th percentile was coded as 1).

d Defined as the number of objective indicators of social risk (i.e., unemployment/unable to work/early retirement; low education of both parents as a proxy for low socioeconomic status; foreign born/second generation migration; living alone/alone with kids; being single; range social risk index: 0–6).

e Based on the following item “Do you already use mHealth apps (e.g., to relax or increase physical activity)?” binary outcome variable: answering “never” was coded as 0, whereas answering “very rarely,” “rarely,” “occasionally,” “often,” or “very often” was coded as 1.

f Based on the following item “Do you think that an mHealth app could help you deal better with the corona situation?” this item was rated on a 5-point Likert scale and dichotomized (with ratings of 1 = “strongly disagree” to 3 = “neither/nor” were coded as 0, and ratings of 4 = “agree” and 5 = “strongly agree” were coded as 1).

### Social isolation and COVID-19-related preoccupation, worries, and anxiety by psychological distress

**Figure 1.**
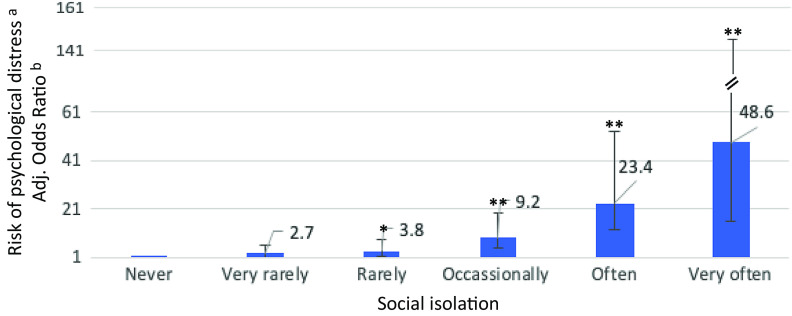
Associations of social isolation with psychological distress. *Notes:* Odds ratios and 95% confidence intervals are shown. *p < 0.05; **p < 0.001. ^a^K10 cutoff of >19 has been used to index presence vs. absence of any mild, moderate, or severe psychological distress as the outcome variable. ^b^Model adjusted for age, gender, educational level, migrant/ethnic minority group position, and employment status.

Individuals who reported subjective experiences of social isolation, lack of company, and COVID-19-related worries and anxiety were more likely to experience psychological distress during the COVID-19 pandemic. As shown in Table 2, there was evidence for dose–response relationships in that psychological distress was progressively more likely to occur as levels of reported social isolation, lack of company, and COVID-19-related worries and anxiety increased. For example, those who reported to be “rarely,” “occasionally,” “often,” and “very often” socially isolated were around 4, 9, 22, and 42 times, respectively, more likely to experience psychological distress (adjusted odds ratio [aOR] 3.7, 95% confidence interval [CI] 1.7–8.1, *p* = 0.006; aOR 9.1, CI 4.3–19.1, *p* < 0.001; aOR 22.2, CI 9.8–50.2, *p* < 0.001; aOR 42.3, CI 14.1–126.8, *p* < 0.001; respectively) as compared to those who reported to be “never” socially isolated (see Figure 1).

**Table 2.** Social isolation, lack of company, cognitive preoccupation, worrying, anxiety, and social risk index by psychological distress during COVID-19 pandemic (*N* = 666).

Exposure					Outcome: Presence of mental distress during COVID-19 pandemic^a^						
		Total, *n* (%)	Presence, *n* (%)	Absence, *n* (%)	Model 1^b^		Model 2^c^		Model 3^d^		
					OR (95% CI)	*p*	OR (95% CI)	*p*	Adj. OR (95% CI)	*p*	*p*FWE
Social isolation											
	Never	61 (9.2)	10 (16.4)	51 (83.6)	1		1		1		
	Very rarely	65 (9.8)	21 (32.3)	44 (67.7)	2.4 (1.0–5.7)	0.041	2.5 (1.0–5.8)	0.039	2.6 (1.1–6.2)	0.029	0.205
	Rarely	135 (20.3)	56 (41.5)	79 (58.5)	3.6 (1.7–7.7)	<0.001	3.6 (1.7–7.8)	<0.001	3.7 (1.7–8.1)	<0.001	0.006
	Occasionally	212 (31.8)	132 (62.3)	80 (37.7)	8.4 (4.0–17.5)	<0.001	8.4 (4.0–17.5)	<0.001	9.1 (4.3–19.1)	<0.001	<0.001
	Often	136 (20.4)	109 (80.2)	27 (19.8)	20.6 (9.3–45.7)	<0.001	21.0 (9.4–46.8)	<0.001	22.2 (9.8–50.2)	<0.001	<0.001
	Very often	57 (8.6)	51 (89.5)	6 (10.5)	43.4 (14.7–128.2)	<0.001	43.8 (14.7–130.2)	<0.001	42.3 (14.1–126.8)	<0.001	<0.001
Lack of company											
	Never	34 (5.1)	4 (11.8)	30 (88.2)	1		1		1		
	Very rarely	43 (6.5)	20 (46.5)	23 (53.5)	6.5 (2.0–21.7)	0.002	6.5 (2.0–21.7)	0.002	7.7 (2.2–26.7)	0.001	0.009
	Rarely	81 (12.2)	37 (45.7)	44 (54.3)	6.3 (2.0–19.5)	0.001	6.3 (2.0–19.5)	0.001	7.7 (2.4–24.8)	<0.001	0.004
	Occasionally	245 (36.8)	125 (51.0)	120 (49.0)	7.8 (2.7–22.8)	<0.001	7.7 (2.6–22.6)	<0.001	9.3 (3.1–28.5)	<0.001	<0.001
	Often	178 (26.7)	124 (69.7)	54 (30.3)	17.2 (5.8–51.3)	<0.001	16.9 (5.7–50.4)	<0.001	21.0 (6.7–65.3)	<0.001	<0.001
	Very often	85 (12.3)	69 (81.2)	16 (18.8)	32.3 (10.0–104.9)	<0.001	31.7 (9.7–103.4)	<0.001	38.0 (11.2–128.3)	<0.001	<0.001
Cognitive preoccupation with COVID-19											
The novel coronavirus is something I…											
	…never think of	20 (3.0)	5 (25.0)	15 (75.0)	1		1		1		
	…do not think about very often	115 (17.3)	46 (40.0)	69 (60.0)	2.0 (0.7–5.9)	0.208	1.9 (0.7–5.7)	0.229	2.4 (0.8–7.5)	0.134	0.935
	…think in part	245 (36.8)	144 (58.8)	101 (41.2)	4.3 (1.5–12.1)	0.006	4.1 (1.4–11.7)	0.008	5.2 (1.7–15.9)	0.004	0.025
	…think about a lot	245 (36.8)	157 (64.1)	88 (35.9)	5.4 (1.9–15.2)	0.002	5.1 (1.8–14.5)	0.003	6.6 (2.2–20.1)	0.001	0.007
	…keep thinking about	41 (6.2)	27 (65.9)	14 (34.1)	5.8 (1.7–19.2)	0.004	5.4 (1.6–18.0)	0.006	7.1 (2.0–25.2)	0.003	0.018
COVID-19-related anxiety											
The novel coronavirus is…											
	…not scary at all	105 (15.8)	31 (29.5)	74 (70.5)	1		1		1		
	…rather not scary	187 (28.1)	92 (49.2)	95 (50.8)	2.3 (1.4–3.8)	0.001	2.3 (1.4–3.8)	0.001	2.5 (1.5–4.1)	<0.001	0.006
	…partly scary	219 (32.9)	140 (63.9)	79 (36.1)	4.2 (2.6–7.0)	<0.001	4.1 (2.5–6.9)	<0.001	4.3 (2.5–7.2)	<0.001	<0.001
	…rather scary	133 (20.0)	97 (72.9)	36 (27.1)	6.4 (3.6–11.3)	<0.001	6.3 (3.6–11.2)	<0.001	6.6 (3.7–11.9)	<0.001	<0.001
	…scary	22 (3.3)	19 (86.4)	3 (13.6)	15.1 (4.2–54.8)	<0.001	14.7 (4.0–53.5)	<0.001	13.8 (3.8–50.6)	<0.001	<0.001
COVID-19-related worrying											
The novel coronavirus is…											
	…not worrying	70 (10.5)	26 (37.1)	44 (62.9)	1		1		1		
	…rather not worrying	142 (21.3)	62 (43.7)	80 (56.3)	1.3 (0.7–2.4)	0.366	1.3 (0.7–2.3)	0.394	1.3 (0.7–2.4)	0.372	1.0
	…partly worrying	207 (31.1)	126 (60.9)	81 (39.1)	2.6 (1.5–4.6)	0.001	2.6 (1.5–4.5)	0.001	2.5 (1.4–4.5)	0.001	0.010
	…rather worrying	207 (31.1)	134 (64.7)	73 (35.3)	3.1 (1.8–5.5)	<0.001	3.0 (1.7–5.3)	<0.001	3.1 (1.7–5.6)	<0.001	<0.001
	…worrying	40 (6.0)	31 (77.5)	9 (22.5)	5.8 (2.4–14.1)	<0.001	5.5 (2.2–13.5)	<0.001	5.6 (2.3–13.8)	<0.001	0.001
COVID-19-related worries^e^											
	<50th percentile	350 (52.6)	140 (36.9)	210 (73.2)	1		1		1		
	≥50th percentile	316 (47.4)	239 (63.1)	77 (26.8)	4.7 (3.3–6.5)	<0.001	4.6 (3.3–6.4)	<0.001	4.5 (3.2–6.3)	<0.001	<0.001
		Mean (SD)									
	Continuous variable	3.6 (1.2)	NA	NA	2.3 (1.9–2.7)	<0.001	2.3 (1.9–2.7)	<0.001	2.2 (1.9–2.7)	<0.001	<0.001

Abbreviations: CI, confidence interval; OR, odds ratio; SD, standard deviation; *p*FWE, family-wise error-corrected *p*-values were computed by multiplying the unadjusted *p*-value by the total number of independent variables (*N* = 7) to adjust significance levels; NA, not applicable.

a K10 cutoff of >19 has been used to index presence vs. absence of any mild, moderate, or severe psychological distress as the outcome variable.

b Unadjusted model.

c Model adjusted for age and gender.

d Model adjusted for age and gender and social risk indicators (i.e., education, migrant/ethnic minority group position, and employment status).

e Based on 10 items asking for potential worries related to the COVID-19 pandemic (e.g., health system overloaded, financial difficulties, and completion of education/school) during the last week rated on a 7-point Likert scale (1 = “no worries at all”; 7 = “a lot of worries”). Items were dichotomized (i.e., median split of the continues variable: <50th percentile was coded as 0 and ≥50th percentile was coded as 1).

### Social risk indicators by psychological distress

We next investigated whether objective indicators of social risk were associated with psychological distress in young individuals during the COVID-19 pandemic. First, we investigated associations of all individual indicators of social risk and migrant/ethnic minority group position with psychological distress. We found that individuals from migrant and ethnic minority groups were more likely to experience psychological distress compared to those from the ethnic majority group (aOR 1.7, CI 1.2–2.4, *p* = 0.041). However, after adjustment for multiple testing, there was no evidence that unemployment, being single, lower educational level, parental educational level, or living arrangements were associated with psychological distress (see Table 3). In testing associations between the social risk index and psychological distress, we found that, compared to individuals in whom objective social risk indicators were absent, individuals with two objective indicators were at an increased risk to experience psychological distress during the COVID-19 pandemic (presence of two indicators: aOR 1.9, CI 1.2–3.0, *p* = 0.034). By contrast, there was no strong evidence that, after adjustment for multiple testing, those exposed to only one social risk indicator or three or more indicators were at an increased risk for psychological distress (aOR 1.3, CI 0.9–1.9, *p* = 1.0; aOR 2.1, CI 1.1–3.9, *p* = 0.113, respectively). There was also no evidence of a dose–response relationship.

**Table 3.** Objective social risk indicators by psychological distress during COVID-19 pandemic (*N* = 666).

Exposure				Outcome: Mental distress during COVID-19 pandemic^a^						
	Total, *n* (%)	Presence, *n* (%)	Absence, *n* (%)	Model 1^b^		Model 2^c^		Model 3^d^		
				OR (95% CI)	*p*	Adj. OR (95% CI)	*p*	Adj. OR (95% CI)	*p*	*p*FWE
Employment status^e^										
Absence	634 (95.2)	355 (56.0)	279 (44.0)	1		1		1		
Presence	32 (4.8)	23 (75.0)	8 (25.0)	2.4 (1.0–5.3)	0.039	2.5 (1.1–5.7)	0.029	2.5 (1.1–5.9)	0.032	0.227
Education^f^										
Absence	634 (95.2)	359 (56.6)	275 (43.4)	1		1		1		
Presence	32 (4.8)	20 (62.5)	12 (37.5)	1.3 (0.6–2.7)	0.513	1.3 (0.6–2.8)	0.449	1.1 (0.5–2.4)	0.801	1.0
Migrant/ethnic minority group position^g^										
Absence	505 (75.8)	272 (53.9)	233 (46.1)	1		1		1		
Presence	161 (24.2)	107 (66.5)	54 (33.5)	1.7 (1.2–2.5)	0.005	1.7 (1.2–2.5)	0.005	1.7 (1.2–2.4)	0.006	0.041
Socioeconomic status^h^										
Absence	573 (86.0)	328 (57.2)	245 (42.8)	1		1		1		
Presence	93 (14.0)	51 (54.8)	42 (45.2)	0.9 (0.6–1.4)	0.664	0.9 (0.6–1.4)	0.704	0.8 (0.5–1.3)	0.327	1.0
Living arrangements^i^										
Absence	542 (81.4)	307 (56.6)	235 (43.4)	1		1		1		
Presence	124 (18.6)	72 (58.1)	52 (41.9)	1.1 (0.7–1.6)	0.773	1.1 (0.7–1.6)	0.658	1.1 (0.7–1.7)	0.644	1.0
Relationship status^j^										
Absence	288 (43.2)	151 (52.4)	137 (47.6)	1		1		1		
Presence	378 (56.8)	228 (60.3)	150 (39.7)	1.4 (1.0–1.9)	0.042	1.5 (1.1–2.1)	0.017	1.5 (1.1–2.0)	0.024	0.169
Social risk index^k^										
0	160 (24.0)	79 (49.4)	81 (50.6)	1		1		NA		
1	270 (40.5)	149 (55.2)	121 (44.8)	1.3 (0.9–1.9)	0.244	1.3 (0.9–1.9)	0.223	NA		1.0
2	174 (26.1)	110 (63.2)	64 (36.8)	1.8 (1.1–2.7)	0.011	1.9 (1.2–3.0)	0.005	NA		0.034
3+	62 (9.3)	41 (66.1)	21 (33.9)	2.0 (1.1–3.7)	0.026	2.1 (1.1–3.9)	0.016	NA		0.113

Abbreviations: CI, confidence interval; OR, odds ratio; *p*FWE, family-wise error-corrected *p*-values were computed by multiplying the unadjusted *p*-value by the total number of independent variables (*N* = 7) to adjust significance levels; NA, not applicable.

a K10 cutoff of >19 has been used to index presence vs. absence of any mild, moderate, or severe psychological distress as the outcome variable.

b Unadjusted model.

c Model adjusted for age and gender.

d Model adjusted for age and gender and social risk indicators (i.e., education, migrant/ethnic minority group position, employment status, and depending on depending variable).

e Binary variable: defined as unemployment, unable to work, early retirement (coded as 1) *vs.* other (coded as 0).

f Binary variable: defined as low education level, that is, secondary school qualification as well as no school-leaving qualification (coded as 1) *vs.* other (coded as 0).

g Binary variable: defined as being foreign born or second-generation migrant (coded as 1) *vs.* other (coded as 0).

h Binary variable: defined as low educational level (i.e., secondary school qualification, no school-leaving qualification) of both parents as a proxy for low socioeconomic status (coded as1) *vs.* other (coded as 0).

i Binary variable: defined as living alone or alone with children (coded as 1) *vs.* other (coded as 0).

j Binary variable: defined as being single (coded as 1) *vs.* other (coded as 0).

k Defined as the number of objective indicators of social risk (i.e., unemployment/unable to work/early retirement; low education of both parents as a proxy for low socioeconomic status; foreign born/second generation migration; living alone/alone with kids; being single; range social risk index: 0–6).

### Psychological distress, COVID-19-related preoccupation, worries, anxiety, and social isolation by current mHealth app use

There was some evidence that psychological distress, perceived social isolation, and lack of company, as well as COVID-19-related cognitive preoccupation, worries, and anxiety were associated with current use of mHealth apps (Table 4). For example, those with severe levels of psychological distress were two times more likely to use mHealth apps compared to those without psychological distress (aOR 2.3, CI 1.4–3.6, *p* = 0.007). However, those with mild and moderate levels of psychological distress were as likely to use mHealth apps as those without psychological distress after adjustments for multiple testing. Furthermore, youth who perceived a lack of company were more likely to use mHealth apps (“occasionally”: aOR 4.2, CI 2.0–9.1, *p* = 0.002; “often”: aOR 4.1, CI 1.8–9.0, *p* = 0.004; respectively) as compared to those who reported to “never” experience a lack of company during the COVID-19 pandemic, although some inconsistencies were found. In contrast, there was no evidence that objective indicators of social risk were associated with the use of mHealth apps.

**Table 4.** Current use of mHealth apps by psychological distress, social isolation, lack of company, cognitive preoccupation, worrying, anxiety, and social risk index (*N* = 666).

Exposure					Outcome: Current use of mHealth apps^a^						
		Total, *n* (%)	Yes, *n* (%)	No, *n* (%)	Model 1^b^		Model 2^c^		Model 3^d^		
					OR (95% CI)	*p*	Adj. OR (95% CI)	*p*	Adj. OR (95% CI)	*p*	*p*FWE
Psychological distress^e^											
	None	287 (43.1)	183 (63.8)	104 (36.2)	1		1		1		
	Mild	130 (19.5)	101 (77.7)	29 (22.3)	2.0 (1.2–3.2)	0.005	2.0 (1.2–3.2)	0.006	1.9 (1.2–3.2)	0.008	0.061
	Moderate	104 (15.6)	75 (72.1)	29 (29.9)	1.5 (0.9–2.4)	0.125	1.4 (0.8–2.2)	0.221	1.4 (0.9–2.4)	0.166	1.0
	Severe	145 (21.8)	114 (78.6)	31 (21.4)	2.1 (1.3–3.3)	0.002	2.1 (1.3–3.3)	0.002	2.3 (1.4–3.6)	<0.001	0.007
COVID-19-related worries^f^											
	<50th percentile	350 (52.6)	235 (67.1)	115 (32.9)	1		1		1		
	≥50th percentile	316 (47.4)	238 (75.3)	78 (24.7)	1.5 (1.1–2.1)	0.021	1.5 (1.0–2.1)	0.026	1.5 (1.1–2.2)	0.014	0.114
		Mean (SD)									
	Continuous variable	3.6 (1.2)	NA	NA	1.3 (1.1–1.5)	0.001	1.3 (1.1–1.5)	0.002	1.3 (1.1–1.5)	0.001	0.010
		Total, *n* (%)									
Social isolation											
	Never	61 (9.2)	33 (54.1)	28 (45.9)	1		1		1		
	Very rarely	65 (9.8)	43 (66.2)	22 (33.8)	1.7 (0.8–3.4)	0.168	1.7 (0.8–3.6)	0.142	1.6 (0.8–3.3)	0.211	1.0
	Rarely	135 (20.3)	94 (69.6)	41 (30.4)	1.9 (1.0–3.6)	0.036	2.0 (1.1–3.8)	0.031	1.8 (1.0–3.5)	0.065	0.518
	Occasionally	212 (31.8)	162 (76.4)	50 (23.6)	2.7 (1.5–5.0)	0.001	2.7 (1.5–4.9)	0.001	2.7 (1.4–4.9)	0.002	0.014
	Often	136 (20.4)	106 (77.9)	30 (22.1)	3.0 (1.6–5.7)	0.001	3.0 (1.6–5.8)	<0.001	2.9 (1.5–5.5)	0.002	0.016
	Very often	57 (8.6)	35 (61.4)	22 (38.6)	1.3 (0.6–2.8)	0.423	1.3 (0.6–2.7)	0.517	1.2 (0.6–2.6)	0.614	1.0
Lack of company											
	Never	34 (5.1)	13 (38.2)	21 (61.8)	1		1		1		
	Very rarely	43 (6.5)	31 (72.1)	12 (27.9)	4.2 (1.6–10.9)	0.004	4.3 (1.6–11.2)	0.003	3.7 (1.4–10.0)	0.009	0.070
	Rarely	81 (12.2)	56 (69.1)	25 (30.9)	3.6 (1.6–8.4)	0.003	3.7 (1.6–8.6)	0.002	3.3 (1.4–7.7)	0.007	0.057
	Occasionally	245 (36.8)	182 (74.3)	63 (25.7)	4.7 (2.2–9.9)	<0.001	4.6 (2.1–9.7)	<0.001	4.2 (2.0–9.1)	<0.001	0.002
	Often	178 (26.7)	133 (74.7)	45 (25.3)	4.8 (2.2–10.3)	<0.001	4.5 (2.1–9.8)	<0.001	4.1 (1.8–9.0)	<0.001	0.004
	Very often	85 (12.3)	58 (68.2)	27 (31.8)	3.5 (1.5–7.9)	0.003	3.3 (1.4–7.7)	0.006	3.0 (1.3–7.2)	0.010	0.086
Cognitive preoccupation with COVID-19											
The novel coronavirus is something I…											
	…never think of	20 (3.0)	7 (35.0)	13 (65.0)	1		1		1		
	…do not think about very often	115 (17.3)	71 (61.7)	44 (38.3)	3.0 (1.1–8.1)	0.030	2.9 (1.1–7.9)	0.037	2.4 (0.8–6.8)	0.103	0.820
	…think in part	245 (36.8)	180 (73.5)	65 (26.5)	5.1 (2.0–13.5)	0.001	4.9 (1.9–13.0)	0.001	4.2 (1.5–11.5)	0.005	0.042
	…think about a lot	245 (36.8)	188 (76.7)	57 (23.3)	6.1 (2.3–16.1)	<0.001	5.7 (2.2–15.2)	<0.001	4.9 (1.8–13.5)	0.002	0.018
	…keep thinking about	41 (6.2)	27 (65.9)	14 (34.2)	3.6 (1.2–11.0)	0.026	3.2 (1.0–10.0)	0.045	2.6 (0.8–8.6)	0.105	0.840
COVID-19-related anxiety The novel coronavirus is…											
	…not scary at all	105 (15.8)	59 (56.2)	46 (43.8)	1		1		1		
	…rather not scary	187 (28.1)	129 (69.0)	58 (31.0)	1.7 (1.1–2.8)	0.029	1.7 (1.0–2.8)	0.035	1.6 (0.9–2.6)	0.075	0.600
	…partly scary	219 (32.9)	165 (75.3)	54 (24.7)	2.4 (1.5–3.9)	0.001	2.3 (1.4–3.7)	0.001	2.1 (1.3–3.6)	0.003	0.026
	…rather scary	133 (20.0)	105 (79.0)	28 (21.0)	2.9 (1.7–5.2)	<0.001	2.7 (1.5–4.9)	<0.001	2.6 (1.5–4.7)	0.001	0.009
	…scary	22 (3.3)	15 (68.2)	7 (31.8)	1.7 (0.6–4.4)	0.303	1.5 (0.6–4.1)	0.410	1.5 (0.6–4.2)	0.400	1.0
The novel coronavirus is…											
	…not worrying	70 (10.5)	38 (54.3)	32 (45.7)	1		1		1		
	…rather not worrying	142 (21.3)	94 (66.2)	48 (33.8)	1.6 (0.9–3.0)	0.094	1.6 (0.9–2.8)	0.133	1.5 (0.8–2.7)	0.198	1.0
	…partly worrying	207 (31.1)	150 (72.5)	57 (27.5)	2.2 (1.3–3.9)	0.005	2.1 (1.2–3.7)	0.010	2.0 (1.1–3.6)	0.017	0.138
	…rather worrying	207 (31.1)	165 (79.7)	42 (20.3)	3.3 (1.9–5.9)	<0.001	3.2 (1.8–5.7)	<0.001	3.0 (1.6–5.4)	<0.001	0.003
	…worrying	40 (6.0)	26 (65.0)	14 (35.0)	1.6 (0.7–3.5)	0.275	1.3 (0.6–3.0)	0.500	1.3 (0.6–3.0)	0.509	1.0
Social risk index^g^											
	0	160 (24.0)	123 (76.9)	37 (23.1)	1		1		NA		
	1	270 (40.5)	194 (71.9)	76 (28.1)	0.8 (0.5–1.2)	0.253	0.8 (0.5–1.3)	0.422	NA	0.423	1.0
	2	174 (26.1)	117 (67.2)	57 (32.8)	0.6 (0.4–1.0)	0.051	0.7 (0.4–1.1)	0.138	NA	0.138	1.0
	3+	62 (9.3)	39 (62.9)	23 (37.1)	0.5 (0.3–1.0)	0.037	0.5 (0.3–1.0)	0.055	NA	0.080	0.437

Abbreviations: CI, confidence interval; OR, odds ratio; SD, standard deviation*; p*FWE, family-wise error-corrected *p*-values were computed by multiplying the unadjusted *p*-value by the total number of independent variables (*N* = 8) to adjust significance levels; NA, not applicable.

a Based on the following item “Do you already use mHealth apps (e.g., to relax or increase physical activity)?” binary outcome variable: answering “never” was coded as 0, whereas answering “very rarely,” “rarely,” “occasionally,” “often,” or “very often” was coded as 1.

b Unadjusted model.

c Model adjusted for age and gender.

d Model adjusted for age and gender and objective indicators of social risk (e.g., low education, migrant/ethnic minority group position, and unemployment).

e The following K10 cutoffs were used to categorize severity levels of psychological distress: “none” (range score: 10–19); “mild” (range score: 20–24); “moderate” (range score: 25–29); “severe” (range score: 30–50).

f Based on 10 items asking for potential worries related to the COVID-19 pandemic (e.g., health system overloaded, financial difficulties, and completion of education/school) during the last week rated on a 7-point Likert scale (1 = “no worries at all”; 7 = “a lot of worries”). Items were dichotomized (i.e., median split of the continues variable: <50th percentile was coded as 0 and ≥50th percentile was coded as 1).

g Defined as the number of objective indicators of social risk (i.e., unemployment/unable to work/early retirement; low education of both parents as a proxy for low socioeconomic status; foreign born/second generation migration; living alone/alone with kids; being single; range social risk index: 0–6).

### Psychological distress, COVID-19-related worries, anxiety, and social isolation by attitude toward mHealth apps

**Figure 2.**
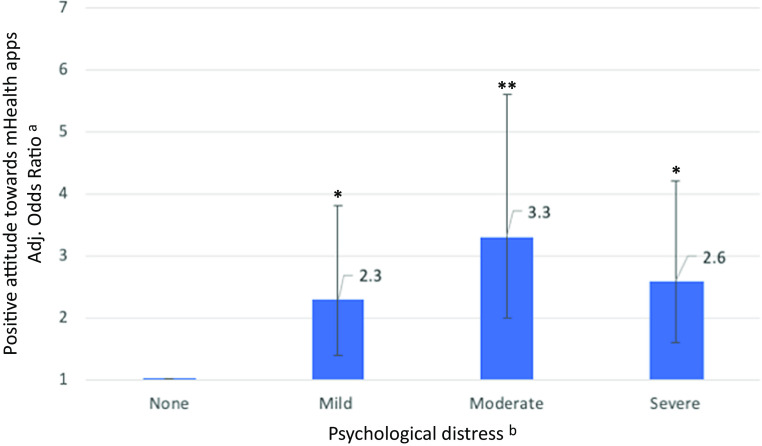
Associations of psychological distress with the positive attitude toward using mHealth apps. *Notes:* Odds ratios and 95% confidence intervals are shown. *p < 0.05; **p < 0.001. ^a^Model adjusted for age and gender and social risk indicators (i.e., education, migrant/ethnic minority group position, and employment status). ^b^The following K10 cutoffs were used to categorize severity levels of psychological distress: “none” (range score: 10–19); “mild” (range score: 20–24); “moderate” (range score: 25–29); “severe” (range score: 30–50).

As shown in Table 5 and Figure 2, individuals who experienced psychological distress were, across all levels of severity, more likely to report a positive attitude toward the use of mHealth apps (mild psychological distress: aOR 2.2, CI 1.34–3.6, *p* = 0.013; moderate: aOR 3.2, CI 1.9–5.4, *p* < 0.001; severe: aOR 2.5, CI 1.6–4.0, *p* = 0.002) than those who did not report psychological distress. Similarly, those with more pronounced COVID-19-related worries (≥50th percentile: aOR 1.8, CI 1.3–2.6, *p* = 0.007), anxiety (“The novel coronavirus is rather scary”: aOR 4.0, CI 2.0–8.0, *p* < 0.001; “The novel coronavirus is scary”: aOR 6.9, CI 2.5–19.5, *p* = 0.002), or high levels of cognitive preoccupation with COVID-19 (“The novel coronavirus is something I keep thinking about”: aOR 10.5, CI 2.1–53.1, *p* = 0.038) were more likely to report a positive attitude toward the use of mHealth apps. However, social isolation, lack of company, and objective indicators of social risk were not associated with individuals’ attitudes toward the use of mHealth apps to address psychosocial consequences of the COVID-19 pandemic after adjustments for multiple testing.

**Table 5.** Attitude toward mHealth apps by psychological distress, social isolation, lack of company, cognitive preoccupation, worrying, anxiety, and social risk index (*N* = 666).

Exposure				Outcome: Attitude toward the use of mHealth apps^a^						
	Total, *n* (%)	Positive, *n* (%)	Neutral/negative, *n* (%)	Model 1^b^		Model 2^c^		Model 3^d^		
				OR (95% CI)	*p*	Adj. OR (95% CI)	*p*	Adj. OR (95% CI)	*p*	*p*FWE
Psychological distress^e^										
None	287 (43.1)	45 (15.7)	242 (84.3)	1		1		1		
Mild	130 (19.5)	39 (30.0)	91 (70.0)	2.3 (1.4–3.8)	0.001	2.3 (1.4–3.8)	<0.001	2.2 (1.3–3.6)	0.002	0.013
Moderate	104 (15.6)	39 (37.5)	65 (62.5)	3.2 (1.9–5.4)	<0.001	3.2 (1.9–5.4)	<0.001	3.2 (1.9–5.4)	<0.001	<0.001
Severe	145 (21.8)	47 (32.4)	98 (67.6)	2.6 (1.6–4.1)	<0.001	2.6 (1.6–4.1)	<0.001	2.5 (1.6–4.0)	<0.001	0.002
COVID-19-related worries^f^										
<50th percentile	350 (52.6)	70 (20.0)	280 (80.0)	1		1		1		
≥50th percentile	316 (47.4)	100 (31.7)	216 (68.3)	1.9 (1.3–2.6)	<0.001	1.9 (1.3–2.7)	<0.001	1.8 (1.3–2.6)	<0.001	0.007
	Mean (SD)									
Continuous	3.6 (1.2)	NA	NA	1.4 (1.2–1.7)	<0.001	1.4 (1.2–1.7)	<0.001	1.4 (1.2–1.7)	<0.001	<0.001
	Total, *n* (%)									
Social isolation										
Never	61 (9.2)	13 (21.3)	48 (78.7)	1		1		1		
Very rarely	65 (9.8)	12 (18.5)	53 (81.5)	0.8 (0.3–2.0)	0.689	0.8 (0.4–2.0)	0.703	0.8 (0.3–2.0)	0.678	1.0
Rarely	135 (20.3)	25 (18.5)	110 (81.5)	0.8 (0.4–1.8)	0.647	0.8 (0.4–1.8)	0.657	0.8 (0.4–1.7)	0.562	1.0
Occasionally	212 (31.8)	51 (24.1)	161 (75.9)	1.2 (0.6–2.3)	0.656	1.2 (0.6–2.4)	0.634	1.2 (0.6–2.4)	0.645	1.0
Often	136 (20.4)	48 (35.3)	88 (64.7)	2.0 (1.0–4.1)	0.052	2.1 (1.0–4.3)	0.042	2.0 (1.0–4.2)	0.052	0.418
Very often	57 (8.6)	21 (36.8)	36 (63.2)	2.2 (1.0–4.9)	0.065	2.3 (1.0–5.1)	0.052	2.1 (0.9–4.8)	0.083	0.663
Lack of company										
Never	34 (5.1)	5 (14.7)	29 (85.3)	1		1		1		
Very rarely	43 (6.5)	10 (23.3)	33 (76.7)	1.8 (0.5–5.7)	0.350	1.8 (0.5–5.9)	0.335	1.7 (0.5–5.8)	0.364	1.0
Rarely	81 (12.2)	14 (17.3)	67 (82.7)	1.2 (0.4–3.7)	0.734	1.2 (0.4–3.7)	0.711	1.2 (0.4–3.8)	0.728	1.0
Occasionally	245 (36.8)	54 (22.0)	191 (78.0)	1.6 (0.6–4.4)	0.330	1.7 (0.6–4.6)	0.306	1.7 (0.6–4.7)	0.302	1.0
Often	178 (26.7)	59 (33.2)	119 (66.9)	2.9 (1.1–7.8)	0.038	3.0 (1.1–8.1)	0.034	3.0 (1.1–8.2)	0.035	0.282
Very often	85 (12.3)	28 (32.9)	57 (67.1)	2.8 (1.0–8.2)	0.051	3.0 (1.0–8.7)	0.041	3.0 (1.0–8.7)	0.045	0.356
Cognitive preoccupation with COVID-19										
The novel coronavirus is something I…										
…never think of	20 (3.0)	2 (10.0)	18 (90.0)	1		1		1		
…do not think about very often	115 (17.3)	11 (9.6)	104 (90.4)	1.0 (0.2–4.7)	0.951	1.0 (0.2–4.8)	0.986	1.0 (0.2–5.2)	0.963	1.0
…think in part	245 (36.8)	56 (22.9)	189 (77.1)	2.7 (0.6–11.8)	0.197	2.8 (0.6–12.5)	0.175	3.1 (0.7–14.0)	0.150	1.0
…think about a lot	245 (36.8)	81 (33.1)	164 (66.9)	4.4 (1.0–19.6)	0.049	4.8 (1.1–21.2)	0.040	5.3 (1.2–24.2)	0.032	0.255
…keep thinking about	41 (6.2)	20 (48.8)	21 (51.2)	8.6 (1.8–41.8)	0.008	9.4 (1.9–46.1)	0.006	10.5 (2.1–53.1)	0.005	0.038
COVID-19-related anxiety										
The novel coronavirus is…										
…not scary at all	105 (15.8)	13 (12.4)	92 (87.6)	1		1		1		
…rather not scary	187 (28.1)	43 (23.0)	144 (77.0)	2.1 (1.1–4.1)	0.029	2.1 (1.1–4.2)	0.027	2.2 (1.1–4.3)	0.026	0.211
…partly scary	219 (32.9)	56 (25.6)	163 (74.4)	2.4 (1.3–4.7)	0.008	2.5 (1.3–4.9)	0.006	2.5 (1.3–4.8)	0.008	0.066
…rather scary	133 (20.0)	47 (35.3)	86 (64.7)	3.9 (2.0–7.6)	<0.001	4.0 (2.0–7.9)	<0.001	4.0 (2.0–8.0)	<0.001	<0.001
…scary	22 (3.3)	11 (50.0)	11 (50.0)	7.1 (2.6–19.6)	<0.001	7.4 (2.6–20.6)	<0.001	6.9 (2.5–19.5)	<0.001	0.002
COVID-19-related worrying										
The novel coronavirus is…										
…not worrying	70 (10.5)	6 (8.6)	64 (91.4)	1		1		1		
…rather not worrying	142 (21.3)	35 (24.7)	107 (75.4)	3.5 (1.4–8.8)	0.008	3.5 (1.4–8.8)	0.008	3.5 (1.4–8.9)	0.008	0.062
…partly worrying	207 (31.1)	50 (24.2)	157 (75.9)	3.4 (1.4–8.3)	0.007	3.5 (1.4–8.5)	0.007	3.4 (1.4–8.4)	0.008	0.063
…rather worrying	207 (31.1)	67 (32.4)	140 (67.6)	5.1 (2.1–12.4)	<0.001	5.3 (2.2–13.0)	<0.001	5.4 (2.2–13.2)	<0.001	0.002
…worrying	40 (6.0)	12 (30.0)	28 (70.0)	4.6 (1.6–13.4)	0.006	4.8 (1.6–14.2)	0.005	4.8 (1.6–14.2)	0.005	0.040
Social risk index^g^										
0	160 (24.0)	37 (23.1)	123 (76.9)	1		1		NA		
1	270 (40.5)	68 (25.2)	202 (74.8)	1.1 (0.7–1.8)	0.631	1.1 (0.7–1.8)	0.562	NA		1.0
2	174 (26.1)	45 (25.9)	129 (74.1)	1.2 (0.7–1.9)	0.562	1.2 (0.7–2.0)	0.511	NA		1.0
3+	62 (9.3)	20 (32.3)	42 (67.7)	1.6 (0.8–3.0)	0.164	1.6 (0.8–3.0)	0.161	NA		1.0

Abbreviations: CI, confidence interval; OR, odds ratio; SD, standard deviation; *p*FWE, family-wise error-corrected *p*-values were computed by multiplying the unadjusted *p*-value by the total number of independent variables (*N* = 8) to adjust significance levels; NA, not applicable.

a Based on the following item “Do you think that an mHealth app could help you deal better with the corona situation?” binary variable: answering “strongly disagree,” “disagree,” or “neither/nor” was coded as 0, whereas answering “agree” or “strongly agree” was coded as 1. Thus, individuals who had a negative or neutral attitude toward using an mHealth app were compared to those with a positive attitude.

b Unadjusted model.

c Model adjusted for age and gender.

d Model adjusted for age and gender and objective indicators of social risk (e.g., low education, migrant/ethnic minority group position, and unemployment).

e The following K10 cutoffs were used to categorize severity levels of psychological distress: “none” (range score: 10–19); “mild” (range score: 20–24); “moderate” (range score: 25–29); “severe” (range score: 30–50).

f Based on 10 items asking for potential worries related to the COVID-19 pandemic (e.g., health system overloaded, financial difficulties, and completion of education/school) during the last week rated on a 7-point Likert scale (1 = “no worries at all”; 7 = “a lot of worries”). Items were dichotomized (i.e., median split of the continues variable: <50th percentile was coded as 0 and ≥50th percentile was coded as 1).

g Defined as the number of objective indicators of social risk (i.e., unemployment/unable to work/early retirement; low education of both parents as a proxy for low socioeconomic status; foreign born/second generation migration; living alone/alone with kids; being single; range social risk index: 0–6).

## Discussion

This study investigated whether social isolation, lack of company, COVID-19-related worries and anxiety, as well as objective social risk indicators were associated with psychological distress during the COVID-19 pandemic in a representative sample of adolescents and young adults during active lockdown in Germany. In addition, associations with current use of, and attitude toward, mHealth apps were investigated. First, there was evidence that social isolation, lack of company, and COVID-19-related cognitive preoccupation, worries, and anxiety were associated with psychological distress. Second, we found evidence of dose–response relationships as psychological distress was progressively more likely to occur as the level of reported social isolation, lack of company, and COVID-19-related preoccupation, anxiety, and worrying increased—although some inconsistencies were observed. Third, an association between migrant/ethnic minority group position and psychological distress was found, while other objective indicators of social risk were not associated with psychological distress. Similarly, associations of levels of the social risk index and psychological distress were inconsistent. Fourth, there was evidence that psychological distress and high levels of COVID-19-related cognitive preoccupation, worries, and anxiety were associated with a more positive attitude toward the use of mHealth apps to help overcome negative consequences of the COVID-19 pandemic. Finally, the actual use of mHealth apps was more likely to be evident in those with severe psychological distress, frequent social isolation and lack of company, as well as COVID-19-related preoccupation, anxiety, and worries, although some inconsistencies were found by levels of respective variables.

An important strength of this study is that findings are based on a representative sample of adolescents and young adults who participated in this survey during active lockdown in Germany. However, several limitations should be taken into account before interpreting reported findings. First, the cross-sectional design of the study did not allow us to investigate temporal order and, thus, we cannot rule out that reverse causality may have operated on our findings and, importantly, the complex nature of investigated constructs and the study design exclude any form of causal inference [48]. Also, as we have not assessed variables before the pandemic, we are not able to disentangle the unique additive effects of the pandemic on reported associations. However, longitudinal cohort studies have found that the prevalence of psychological distress and various mental health conditions was considerably higher during the pandemic as compared to time periods before the pandemic [18,20,23,26,27,45], although some inconsistencies were reported [21]. Also, participants were explicitly asked to report levels of psychological distress *during* the COVID-19 pandemic. Second, the very dynamic development of the pandemic may limit the generalizability of findings to latter stages of the ongoing pandemic or subsequent pandemics. Although strong evidence was found that social isolation, worrying, and other psychosocial factors related to public health measures for minimizing transmission rates were strongly associated with psychological distress, it is possible that the withdrawal of restrictions quickly decreases subjective feelings of social isolation and worrying and, thus, may contribute to a reduction of psychological distress for most individuals [20]. However, the survey was conducted after the peak of new cases per day had occurred during the first wave of the pandemic in Germany and some infection control measures were already beginning to be lifted. Thus, our findings may also underestimate prevalence of psychological distress as compared to moments of strict lockdown and high rates of new cases. That said, mental health outcomes may worsen due to ongoing and expected economic uncertainties and, hence, it may be argued that further negative psychosocial consequences are yet to come. Furthermore, we used a conservative method to minimize type I error rate inflation, which further supports robustness of our findings. Third, some of the indicators used to conceptualize social risk may—although frequently being used in social epidemiological studies—apply to young people only to a limited extent. For instance, living alone may not be perceived as indexing social adversity. Also, some social risk indicators may only be contributing to poor mental health later in life (e.g., lower educational level). Fourth, we used a short screening measure (i.e., K10) to assess psychological distress. As the K10 is arguably largely focusing on depressive symptoms (e.g., feeling hopeless/worthless), other potentially important psychopathological domains (e.g., positive psychotic symptoms) have been largely neglected. Finally, due to time constraints, the study was not preregistered before data collection and data on the psychometric properties of COVID-19-related measures (i.e., COSMO worry scale) are very limited. However, we tested a priori defined hypotheses and findings on internal consistency are reported.

Overall, there is accumulating evidence on the negative consequences of the COVID-19 pandemic on public mental health. A number of cross-sectional and longitudinal cohort studies have found detrimental effects of the pandemic on various mental health domains, including psychological distress, depression, anxiety, and an increase of more distal risk factors such as cannabis and alcohol misuse and loneliness [6–24]. These findings are largely in line with findings from this nationally representative survey, i.e., high levels of social isolation, lack of company, COVID-19-related worrying, and anxiety have been reported and found to impact psychological distress in youth during active lockdown in Germany. Although migrant/ethnic minority group position was found to be associated with psychological distress, we found no evidence that an increased number of social risk indicators was associated with increased levels of psychological distress. Thus, our findings partly differ from other studies which have found that psychosocial consequences of the current pandemic are disproportionally distributed in the society and may especially affect those with more inferior social positions or minority status. However, our findings are in line with findings demonstrating more pronounced effects in youth [23,24] and one study has shown associations between loneliness and COVID-19-related distress [25]. Furthermore, positive attitudes toward the use of mHealth apps to help alleviate the psychosocial consequences of the pandemic were highly prevalent and associated with an objective need (e.g., more severe levels of psychological distress and higher levels of worrying). There have been also other studies which have reported that individuals have a positive attitude toward, and increasingly use, digital interventions during the current COVID-19 pandemic across the whole spectrum of public mental health provision (i.e., mental health promotion, prevention, and treatment of mental disorders) [36–38,49,50], and alterations of telemedicine regulations have been reported [51]. However, mHealth apps provide the opportunity of delivering low-threshold, personalized mental health care in daily life.

The present findings suggest that there is a pressing demand for evidence-based public mental health interventions that aim to specifically target the negative consequences of the COVID-19 pandemic [1]. Digital interventions, including eHealth interventions and mHealth apps, may help to mitigate the negative psychosocial consequences by providing evidence-based information, reliably monitoring symptoms, or delivering intervention components in individual’s daily lives [31,36,37,52,53]. Furthermore, digital interventions may be used to ensure continuity of care in the provision of mental health services in case of repeated outbreaks and lockdowns during the pandemic, and for providing and extending digital interventions to the area of mental health promotion and prevention to mitigate the negative impact of the pandemic especially in youth. Digital mHealth interventions may be particularly suited to help achieve this goal, as they have the potential, once developed and evaluated, to be scaled up and broadly offered at the population level.

To conclude, digital interventions may help to mitigate the negative impact of the COVID-19 pandemic on youth mental health, as there is a subjective demand and objective need. Smartphone-based mHealth apps are particularly suited to provide low-threshold and timely public mental health care in times of physical distancing and quarantine. As the quality of evidence of currently available apps in major app stores is often unknown or very limited [54–59], there is an urgent need to (a) develop and evaluate digital interventions specifically designed to address social isolation and poor mental health to actively prepare for a potential worsening of the current pandemic as well as future health crises, (b) make these evidence-based digital interventions publicly available to improve public mental health, and (c) develop digital strategies for continued mental health care as well as mental health promotion and prevention of mental disorders. Finally, decision-makers and stakeholders in the area of public mental health should work on systematically evaluating currently available digital interventions to support young users to find evidenced-based digital tools, which are most helpful for their individual preferences and current needs [60–62].

## Data Availability

The data that support the findings of this study are available from the corresponding author, UR, upon reasonable request.
